# The gut-immune-brain axis in CNS tumors: Causal roles of microbiota and inflammatory proteins unveiled by Mendelian randomization and single-cell transcriptomics

**DOI:** 10.1097/MD.0000000000050109

**Published:** 2026-08-07

**Authors:** Xianwen Cao, Hui Guo, Kun Wang, Qiang Wu, Xuhan Wang, Junfei Shao

**Affiliations:** a Department of Neurosurgery, Wuxi People’s Hospital Affiliated to Nanjing Medical University, Wuxi, Jiangsu, China.

**Keywords:** central nervous system tumors, circulating inflammatory proteins, gut microbiota, Mendelian randomization

## Abstract

There is a growing number of research suggesting that there is an association between gut microbiota and central nervous system (CNS) tumor. However, the causal relationships and the mediation effects of inflammatory proteins in the associations are unclear. We extracted genetic variants associated with gut microbiota, inflammatory proteins, and 4 subtypes of CNS tumors from published genome-wide association studies and performed a Mendelian randomization analysis to identify potential causal effects. The inverse variance weighted method was used as the main method. Mediation analysis and single-cell RNA-seq analysis were performed to explore the mediation effects and the expression in cells. This study identified 73 gut microbial taxa and 11 inflammatory proteins that were significantly associated with CNS tumors. The inflammatory proteins may act as intermediate mediators in the potential causal association between gut microbiota and 4 CNS tumor subtypes. Mediation analysis suggested that CX3CL1 may partially mediate the relationship between gut microbiota and Glioblastoma, while Eotaxin, CSF-1, IL-15RA and the other 5 cytokines may serve as subtype-specific potential mediators for the remaining 3 tumor types. Our research supports a hypothesized “gut-immune-brain” axis that may mediate the effects of gut microbiota on different CNS tumor subtypes, with distinct immune proteins implicated for each. These findings strongly suggest potential targets for microbiome therapy and immune therapy, though the underlying mechanistic links require experimental validation.

## Graphical abstract

**Figure:**
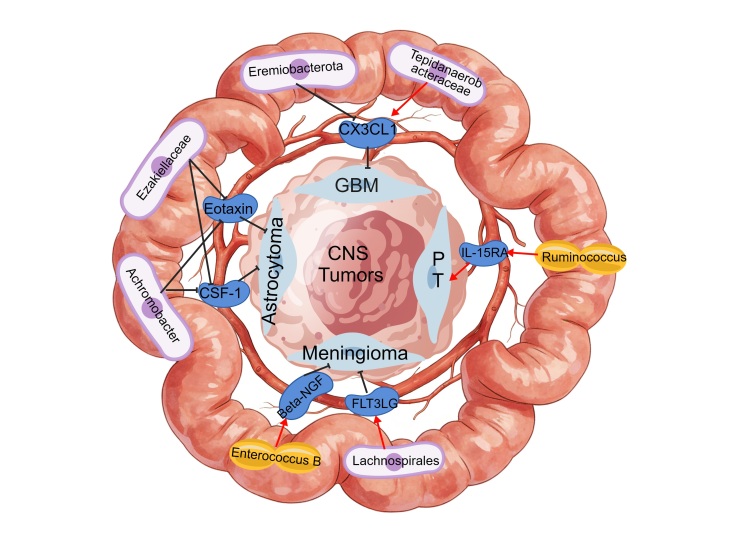

## 1. Introduction

Central nervous system (CNS) tumors are among the deadliest and most disabling cancers, with a complicated pathogenesis and few effective therapies.^[1]^ Primary CNS tumors include glioblastoma (GBM), astrocytoma, pituitary tumors (PT), and meningioma. Epidemiological evidence has indicated that dysbiosis in environmental factors and immune systems may be responsible for the largely unexplained genetic background of the tumorigenesis process, but the details of the specific pathways are unclear. For example, the five-year survival rate of GBM is <10%, and thus clarifying the novel mechanisms is urgently needed to facilitate targeted interventions.^[2]^

The human gut microbiota, the largest symbiotic ecosystem in humans in terms of numbers, has a close and continuous bidirectional interaction with the CNS, also called the “gut-brain axis.”^[3]^ The latest studies found that microbial metabolites, such as short-chain fatty acids (SCFAs) and bile acids, could affect the blood-brain barrier (BBB) permeability mediated by tight junctions, microglia maturation and function, and meninges and neuroinflammation.^[4,5]^ Alterations in specific microbial taxa have been observed in neurodegenerative diseases,^[6]^ but whether gut microbiota directly contributes to CNS tumorigenesis, particularly through systemic inflammatory pathways, requires causal evidence.

Inflammatory inflammatory proteins (e.g., IL-6, TNF-α) are key regulators of the tumor microenvironment (TME), promoting angiogenesis, immune evasion, and oncogenic signaling.^[7,8]^ Prospective cohort studies associate peripheral inflammatory markers with brain tumor-risk,^[9]^ though observational designs cannot exclude reverse causality. Whether inflammatory proteins act as intermediaries in how gut microbiota influences CNS tumors is still an important scientific issue that needs to be addressed.

Genome-Wide Association Study (GWAS) has pinpointed a great many genetic variants associated with complex diseases through the analysis of genome-wide associations across large populations.^[10]^ Publicly accessible GWAS summary statistics related to gut microbiota, circulating inflammatory proteins, and CNS tumors serve as instrumental variables (IVs) in this study. Mendelian randomization (MR) is a commonly used analytical method in the field of genetic epidemiology. In MR studies, researchers use genetic variations as IVs to assess whether there is a causal effect between exposure factors and outcomes, as well as the magnitude of such an effect. They facilitate causal inference through genetic proxies, while addressing constraints associated with sample size and measurement accuracy.^[11–13]^

In this research, a two-sample MR approach was applied to thoroughly investigate the causal associations between 473 gut microbial traits, 91 circulatory inflammatory proteins, and 4 CNS tumors. We performed mediation analysis to examine whether inflammatory proteins are mediators in the gut microbiota–tumor axis. The reverse causality of primary MR findings was validated using bidirectional MR analysis. We provide pioneering genetic evidence on the “microbiota-immune-tumor” axis.

## 2. Study design

**Figure 1.**
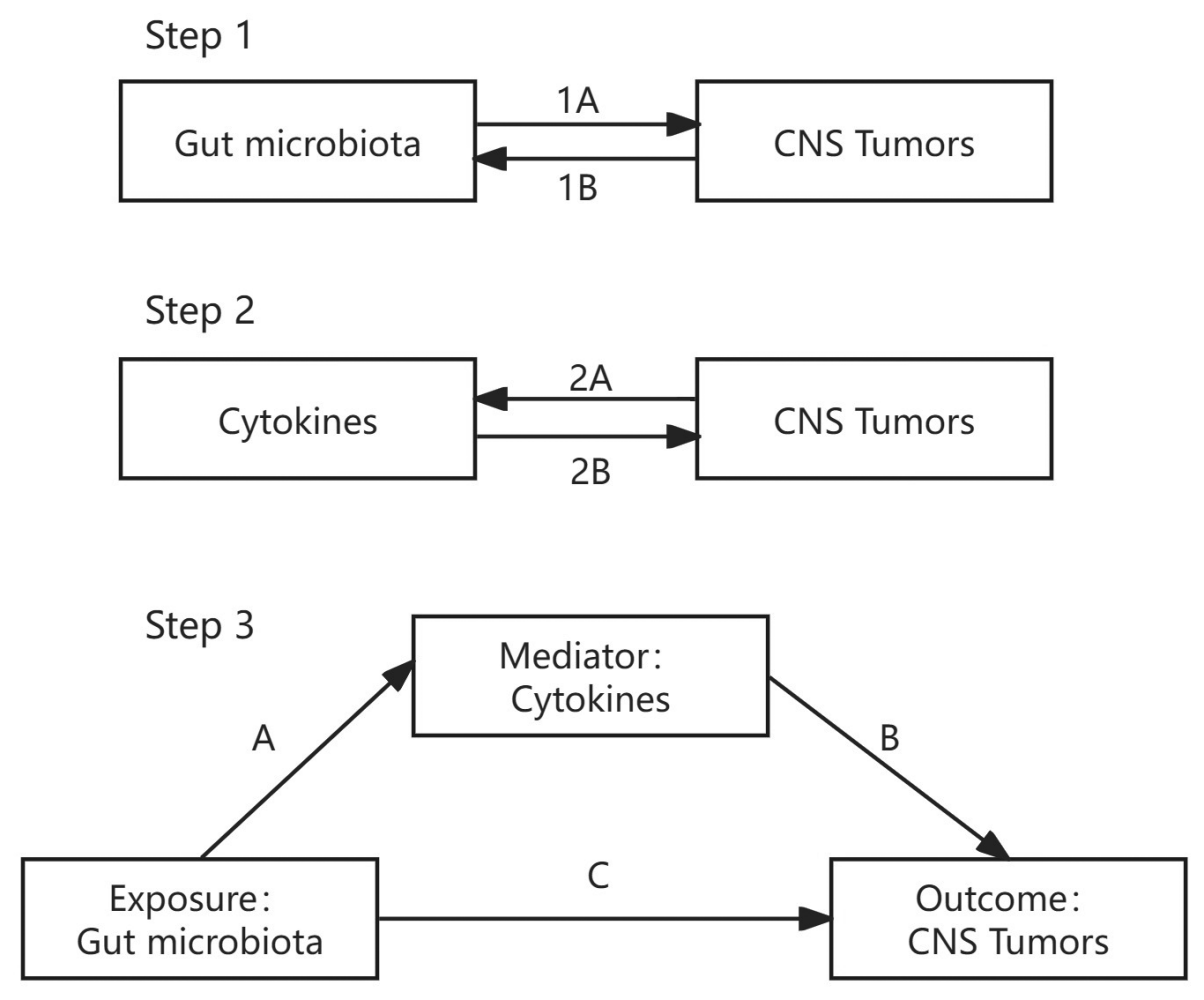
Study overview. Step 1A delineates the unidirectional causal pathway where gut microbiota exerts an effect on CNS Tumors. Step 1B, conversely, captures the bidirectional causal interplay between gut microbiota and CNS tumors, highlighting reciprocal influences. Step 2A maps out how inflammatory proteins drive a causal effect toward CNS tumors, while Step 2B reveals the bidirectional causal dynamics operating between inflammatory proteins and CNS tumors. Step 3 undertakes a mediational exploration: within the gut microbiota - to - CNS tumors axis, path C quantifies the total causal reach of gut microbiota on CNS tumors; path B isolates inflammatory proteins’ direct causal imprint on CNS tumors; and path A pinpoints gut microbiota’s upstream causal shaping of inflammatory proteins.

The framework of this study was illustrated in Figure 1 and comprised 3 main parts: the causal analysis of 473 gut microbiota with 4 CNS tumors (Step 1A); the causal analysis of 91 circulatory inflammatory proteins with 4 CNS tumors (Step 2A); and mediation analysis to investigate the role of inflammatory proteins in the causal effect of gut microbiota on CNS tumors (Step 3). We acquired single-nucleotide polymorphisms (SNPs) as IVs. MR analysis satisfied 3 main assumptions: IVs were strongly associated with exposures; IVs had no association with confounders of exposure-outcome associations; IVs had no direct influence on study outcomes and only affected study outcomes via exposures. The analysis strictly followed the guideline for Strengthening the Reporting of Observational studies in Epidemiology using Mendelian Randomization (STROBE-MR) (Table S1, Supplemental Digital Content 1).^[14]^

## 3. Data source

GWAS data on gut microbiota were derived from the dataset published by Kurilshikov et al which includes 473 gut microbial features and is based on 18,340 individuals.^[15]^ Data on 91 circulating inflammatory proteins came from a meta-analysis covering 11 cohorts, involving a total of 14,824 European participants.^[16]^ All these GWAS data, spanning the accession ranges from GCST90032172 to GCST90032644 for gut microbiota and from GCST90274758 to GCST90274848 for inflammatory proteins, are openly accessible in the NHGRI-EBI Catalog.

Summary GWAS data related to GBM, astrocytoma, PT, and meningioma were obtained from version 12 of the FinnGen consortium (https://r12.risteys.finngen.fi/). Operating as a prospective cohort investigation, the FinnGen consortium pinpointed CNS tumor cases by utilizing International Classification of Diseases diagnostic codes. Genetic information corresponding to each of the 4 CNS tumor categories was separately acquired from the FinnGen database. Further specifics are outlined in Table S2, Supplemental Digital Content 2.

In order to clarify the cellular distribution of the main mediators (i.e., CX3CL1 and its receptor CX3CR1) in the TME of GBM, we conducted a secondary analysis using the publicly accessible dataset of Single-Cell RNA Sequencing (scRNA-seq) (GSE131928) from the GEO database (https://www.ncbi.nlm.nih.gov/geo/). GEO, a public functional genomics data repository, contains high-throughput gene expression data and related metadata, and guarantees the accessibility and reproducibility of the dataset used in this study.

This study was a secondary analysis of publicly available GWAS summary statistics. Ethical approval was obtained for all original GWAS studies. Notably, because this study did not include any individual-level data, no additional approval from an ethics committee was needed.

## 4. IV selection

First, we tried to extract gut microbiota-related SNPs with the threshold of *P* < 5 × 10^−6^. However, valid SNPs were lacking when referenced to the screening threshold. In order to yield an adequate SNP panel for downstream analyses, we selected gut microbiota-related SNPs with a threshold of *P* < 1 × 10^−5^. Second, linkage disequilibrium loci were excluded, and we retained gut microbiota-related SNPs with strict linkage disequilibrium thresholds as follows: pairwise correlation coefficient *r*^2^ < 0.001, physical distance >10,000 kb.^[17]^ Last, an important step in MR analysis was to make sure that the direction of the SNP’s effects on the exposure and on the outcome corresponded to the same allele. After harmonizing the outcome data, palindromic SNPs were excluded; in other words, we eliminated SNPs with A/T or G/C alleles. We also collected related details from every SNP: the position on the chromosome, the effect allele, the non-effect allele, the effect allele frequency, the effect (β), the standard error for β, and the corresponding *P*-value. Finally, the explained variance (R2) and *F*-statistics were calculated to ensure solid correlations between all IVs and the exposure. If the *F*-statistics of a SNP is <10, it can be considered a weak instrument.^[18]^

## 5. MR analysis

### 5.1. Main MR analysis

For main MR analyses of the causal effects of gut microbiota/inflammatory proteins on CNS tumors, we conducted two-sample MR analysis (detailed in Steps 1A and 2A; Fig. 1) using 5 approaches: Inverse Variance Weighted (IVW),^[19]^ Weighted Mode,^[20]^ MR-Egger,^[21]^ Weighted Median,^[22]^ and Simple Mode.^[23]^ In these steps, the IVW method was taken as the principal one, and results are reported as odds ratios (ORs) and 95% confidence intervals (CIs). The IVW *P*-value threshold determined statistical significance. Our results were considered statistically significant based on the following 2 criteria: the IVW *P*-value was <0.05, and the results of IVW and MR-Egger were directionally concordant.

### 5.2. Mediation analysis

We detected significant causal effects of gut microbial and inflammatory proteins on CNS tumors, focusing on the mediation analyses. We started by evaluating the impact of gut microbiota on inflammatory proteins. This portion of the analysis is outlined in Step 3 (Fig. 1, path a). Then, we performed mediation analyses to assess the extent to which inflammatory proteins participate in the relationship between gut microbiota and CNS tumors, with the aim of clarifying their potential intermediate roles.

### 5.3. Bidirectional causality analysis

In order to assess the potential influence of reverse causation, we set CNS tumors as the “exposure” and gut microbiota/inflammatory proteins as the “outcomes’ (detailed in Steps 1B and 2B; Fig. 1) for this part of the analysis. Here, we selected SNPs with a significant association with CNS tumors (*P*-value <1 × 10^−5^) as IVs.

### 5.4. Statistical analysis and multiple testing correction

We performed 3 types of sensitivity analyses. First, we used Cochran’s *Q* tests to assess heterogeneity^[24]^ between causal estimates derived from different SNPs, and scatterplots to graphically view SNPs, exposure, and outcome. Second, leave-one-out analyses were performed by excluding each alternative SNP and recalculating the results using the remaining SNPs. Third, we applied MR-PRESSO and MR-Egger regression methods to identify and correct for horizontal pleiotropy.^[25,26]^ In particular, we used MR-PRESSO outlier tests for the detection of horizontal pleiotropy by excluding outlier SNPs. Furthermore, we utilized Benjamini-Hochberg False Discovery Rate (FDR) correction for multiple testing, and FDR-adjusted *P*-values (pFDR) <0.05 were considered statistically significant. Results with pFDR ≥0.05 were eliminated from our summary results to reduce the chance of reporting false-positive associations, with the aim of guaranteeing the authenticity of the relationships between exposure and outcome.

All analyses were executed in R version v4.3.2. The “TwoSampleMR” package was utilized for MR analyses, and “MRPRESSO” was used for pleiotropy assessment.

## 6. Results

### 6.1. IVs selection

A total of 224,478,166,728 SNPs were identified to be associated with 473 gut microbial taxa. Using a significance threshold of *P* < 1 × 10^−5^, 9281 of these SNPs were further screened out and selected as IVs, with all detailed information of these SNPs available in Table S3, Supplemental Digital Content 3. Additionally, 2808 SNPs linked to 41 inflammatory proteins (with *P* < 1 × 10^−5^) were identified and included as IVs, and all relevant details of these SNPs can be found in Table S4, Supplemental Digital Content 4.

### 6.2. Causal relationships between gut Microbiota, inflammatory proteins, and CNS tumors

**Figure 2.**
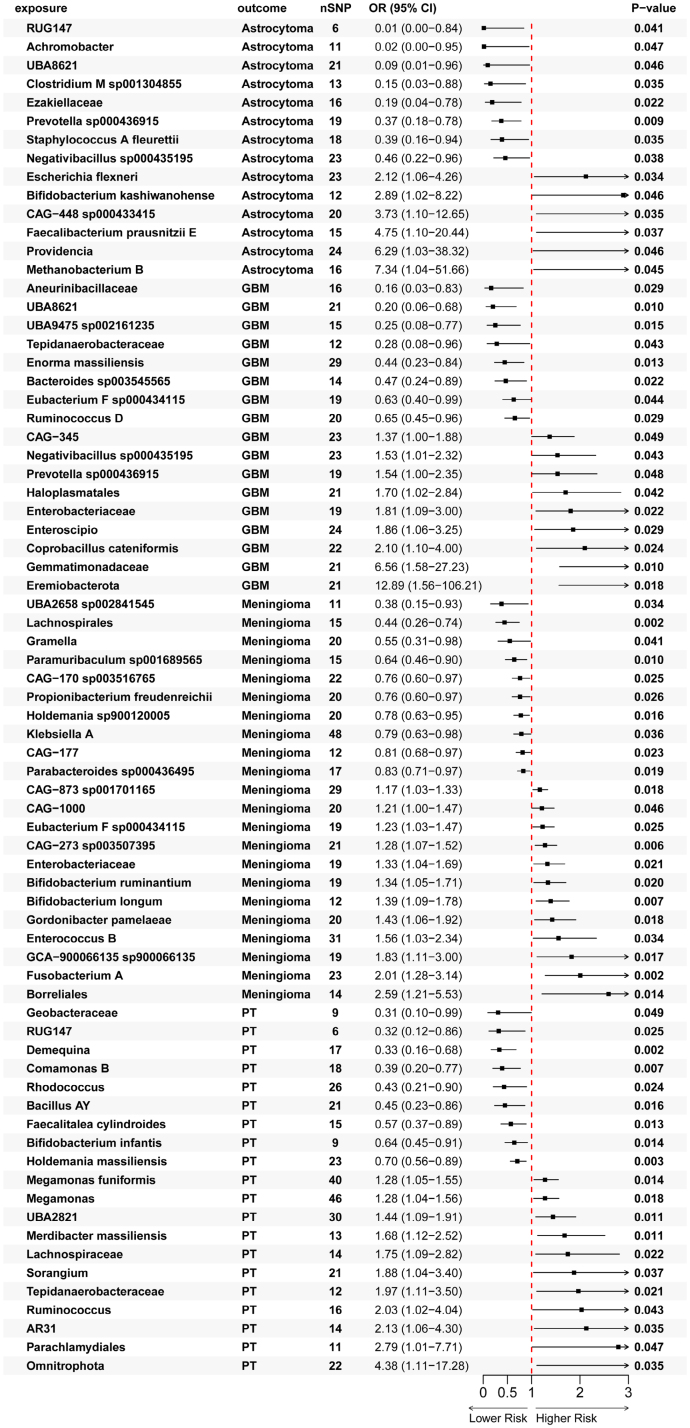
Mendelian randomization results of the associations between gut microbiota taxa and 4 subtypes of central nervous system tumors.

**Figure 3.**
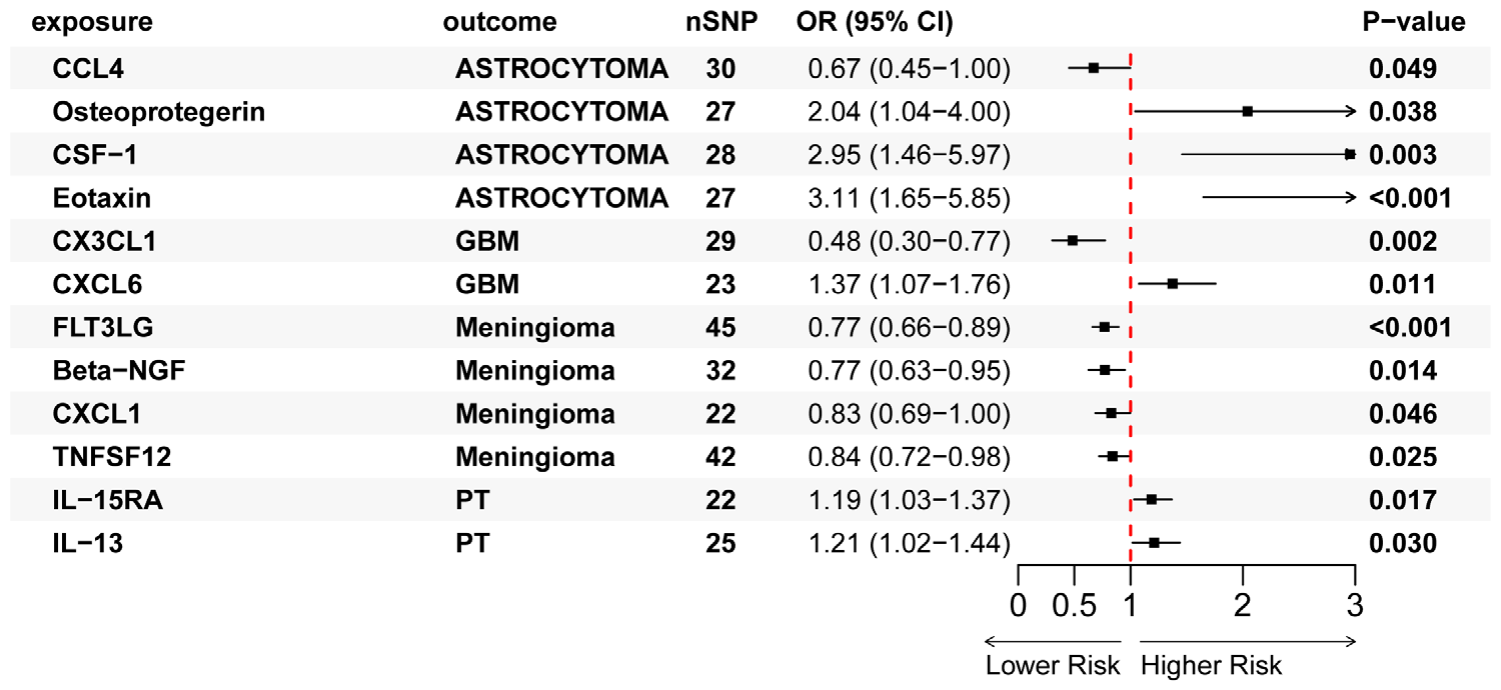
MR results of the associations between inflammatory proteins and 4 subtypes of central nervous system tumors. CI = confidence interval, OR = odds ratio, SNP = single-nucleotide polymorphism.

After MR analysis and FDR correction (pFDR < 0.05), we excluded all association signals with pFDR ≥0.05. Ultimately, we identified 73 gut microbial taxa and 11 circulating inflammatory proteins that show evidence consistent with potential causal associations with 4 CNS tumor subtypes in MR analyses, which were ranked by effect size (Figs. 2, 3; Tables S5, S6 and S7, Supplemental Digital Content 5). Subsequent analyses focused on these rigorously filtered associations, and based on the magnitude of effect sizes and biological plausibility, we further determined the core candidates for each tumor subtype.

### 6.3. Astrocytoma

Astrocytoma had significant causal associations with 14 gut microbial taxa, with relevant statistical data and visualizations in Table S5, Supplemental Digital Content 5 and Figure 2 (sorted by OR value in descending order); Table S6, Supplemental Digital Content 6 provided SNP details of these 14 taxa. Among them, 8 were tumor-risk taxa and 6 were protective, with 4 exhibiting the most prominent effects: two strongly protective taxa (*RUG147* and *Achromobacter*) and 2 high-risk taxa (*Methanobacterium B* and *Providencia*). Specifically, RUG147 (OR = 0.01, 95% CI: 0.00–0.84, *P* = .041) and Achromobacter (OR = 0.02, 95% CI: 0.00–0.95, *P* = .047) had OR < 0.05 and *P* < .05. Previous studies confirmed Achromobacter, enriched by metformin, enhances antitumor effects in colorectal cancer (CRC) models,^[27]^ supporting its protective role. *Methanobacterium B* (OR = 7.34, 95% CI: 1.04–51.66, pFDR = 0.028) and *Providencia* (OR = 6.29, 95% CI: 1.03–38.32, pFDR = 0.035) had OR > 5 and *P* < .05; notably, *Providencia* (an opportunistic pathogen) was reported to impair intestinal barrier function and increase permeability,^[28]^ consistent with its tumor-promoting effect here, implying a potential “intestinal barrier disruption–systemic inflammation–tumor progression” pathway. It should be noted that despite *RUG147*’s strongest protective effect (OR = 0.01), only 6 SNPs were used as IVs for its MR analysis (below the common MR threshold of ≥10 SNPs), risking insufficient statistical power; its reliability requires further verification with larger samples or functional experiments.

As depicted in Figure 3, 4 inflammatory proteins had significant associations with astrocytoma risk, with all relevant detailed data available in Table S7, Supplemental Digital Content 7. Osteoprotegerin, CSF-1, and Eotaxin were linked to heightened risk, while CCL4 was associated with reduced risk. Among these, Eotaxin stood out as the most impactful pro-tumor inflammatory protein, exhibiting a substantially elevated risk effect with an OR of 3.11 (95% CI: 1.65–5.88, *P* < .001). Eotaxin is well-documented to drive the progression of multiple solid tumors, including CRC and ovarian cancer.^[29,30]^ Meanwhile, it is reported that serum Eotaxin levels are significantly elevated in glioma patients,^[8]^ which is consistent with our findings.

### 6.4. GBM

GBM had significant causal associations with 17 gut microbial taxa, with relevant statistical data and visualizations in Table S5, Supplemental Digital Content 5 and Figure 2; Table S6, Supplemental Digital Content 6 provided SNP details of these 17 taxa. Among them, 9 were tumor-risk taxa, and 8 were protective, with 3 exhibiting the most prominent effects: one strongly protective taxon (*Aneurinibacillaceae*) and 2 high-risk taxa (*Gemmatimonadaceae* and *Eremiobacterota*). Specifically, *Aneurinibacillaceae*: the strongest protective factor, with an OR < 0.2 (95% CI: 0.03–0.83, *P* = .029) and reached statistical significance. In contrast, the high-risk taxa *Gemmatimonadaceae* (OR = 6.56, 95% CI: 1.58–27.23, *P* = .010) and *Eremiobacterota* (OR = 12.89, 95% CI: 1.56–106.21, *P *= .018) both had OR > 5. Notably, *Gemmatimonadaceae* has been reported to show increased abundance in the vagina of cervical cancer patients undergoing radiation therapy, and its DNA was detected in lung cancer patients receiving nivolumab treatment, with associations noted between its presence and tumor progression.^[31,32]^This aligns with its strong tumor-promoting effect in GBM observed in our study, suggesting a potential conserved pro-tumor role of Gemmatimonadaceae across different cancer types and treatment backgrounds.

As shown in Figure 3, 2 inflammatory proteins were significantly associated with GBM risk (Table S7, Supplemental Digital Content 7). CX3CL1 (Fractalkine) had a protective effect with OR = 0.48, while CXCL6 (GCP-2) was linked to an increased risk with OR = 1.37.

### 6.5. Meningioma

Twenty-two gut microbial taxa showed a connection to meningioma, and relevant findings are documented in Table S5, Supplemental Digital Content 5, as well as Figure 2. For detailed SNP-related information, refer to Table S6, Supplemental Digital Content 6.

Compared with astrocytoma and GBM, the protective and risk effects of gut microbiota on meningioma were less pronounced: among the associated taxa, we identified 1 key protective taxon and 2 key risk taxa for focused analysis. Specifically, the protective taxon was *UBA2658 sp002841545*, with an OR of 0.38 (95% CI: 0.15–0.93, *P* = .034); the risk taxa were Borreliales (OR = 2.59, 95% CI: 1.21–5.53, *P* = .014) and *Fusobacterium A* (OR = 2.01, 95% CI: 1.28–3.14, *P* = .002). Notably, while direct studies on *Fusobacterium A* remain scarce, its congener *Fusobacterium nucleatum* has been well-documented to promote the development of CRC,^[33]^ primarily by enhancing tumor cell proliferation and inhibiting antitumor immunity. The tumor-promoting effect of *Fusobacterium A* on meningioma observed in our study provides a novel extension of this genus’ role in carcinogenesis, which requires further validation.

Four inflammatory proteins were significantly linked to meningioma risk (Fig. 3 and Table S7, Supplemental Digital Content 7), all showing protective effects: FLT3LG, Beta-NGF, CXCL1, and TNFSF12, with their protective impacts being relatively weak and OR values ranging from 0.77 to 0.84.

### 6.6. PT

Twenty gut microbial taxa were linked to PT, with supporting data available in Table S5, Supplemental Digital Content 5 and Figure 2. Detailed SNP information is provided in Table S6, Supplemental Digital Content 6.

Similar to meningioma, the protective and risk effects of gut microbiota on PT were less pronounced compared with astrocytoma and GBM. Among these associated taxa, *Geobacteraceae* and *Omnitrophota* emerged as the strongest protective and risk factors, respectively. Specifically, *Geobacteraceae* exhibited a protective effect with an OR of 0.31 (95% CI: 0.10–0.99, *P* = .049), while *Omnitrophota* showed a risk effect with an OR of 4.36 (95% CI: 1.11–17.28, *P* = .035). Regrettably, no existing literature has reported associations between these 2 taxa and PT, and their specific roles in PT pathogenesis remain unelucidated.

Two inflammatory proteins were significantly associated with PT risk (Fig. 3 and Table S7, Supplemental Digital Content 7), both of which were linked to an increased risk and acted as weak risk factors: IL-15RA and IL-13.

### 6.7. Sensitivity analyses and multiple testing correction

MR-Egger regression intercept tests showed no signs of directional pleiotropy (*P* > .05). At the same time, supplementary evidence that horizontal pleiotropy does not exist was found in the MR-PRESSO analyses (*P* > .05), and all related details are displayed in Table S8, Supplemental Digital Content 8. Furthermore, the results of Cochran’s *Q* tests, which are also documented in Table S8, Supplemental Digital Content 8, indicated that there was no statistically significant heterogeneity (*P* > .05). By using the Benjamini-Hochberg FDR correction, the IVW results were adjusted for multiple testing. All of the aforementioned significant connections remained robust following the FDR adjustment (pFDR < 0.05), and all related factual statistics are listed in Table S8, Supplemental Digital Content 8.

We performed Leave-one-out analyses for each of the 73 gut microbial taxa and 11 inflammatory proteins that we found to have causal relationships with CNS tumors, which essentially verified the stability of our results, that is, the null line was outside the overall CIs for every exposure-outcome pair tested, demonstrating that no SNP disproportionately influenced the observed causal association (see Supplementary Fig. S1, S2, S3 and S4, Supplemental Digital Content 9, S13, S14, S15 and S16, Supplemental Digital Content 28). The scatter plots (Supplementary Fig. S5, S6, S7 and S8, Supplemental Digital Content 10, S17, S18, S19 and S20, Supplemental Digital Content 32) and forest plots (Supplementary Fig. S9, S10, S11 and S12, Supplemental Digital Content 11, S21, S22, S23 and S24, Supplemental Digital Content 36) for each exposure-outcome pair further illustrate the individual SNP effects and the overall MR estimates, confirming the robustness of the associations.

### 6.8. Bidirectional causal links between CNS tumors, gut microbiota, and inflammatory proteins

Reverse MR analyses revealed bidirectional relationships: Astrocytoma affected 14 gut microbial taxa (with no observed effects on inflammatory proteins); GBM influenced 7 gut microbial taxa and 5 inflammatory proteins; Meningioma impacted 66 gut microbial taxa and 1 cytokine; and PT modified 40 gut microbial taxa and 6 inflammatory proteins. All these effects were modest, with OR values ranging from 0.93 to 1.07 *(P* < .05) (Fig. S49–S50, Supplemental Digital Content 12). It is important to note that ORs near 1 do not inherently indicate biological irrelevance, as they may partially reflect limited statistical power in the reverse direction: consistent with the smaller number of SNPs selected for CNS tumors (*P* < 1 × 10^−5^) compared to gut microbiota and inflammatory proteins, which could constrain the ability to detect stronger causal effects. The corresponding sensitivity analyses: including leave-one-out analyses (Fig. S25, S26, S27 and S28, Supplemental Digital Content 13, S37, S38, S39 and S40, Supplemental Digital Content 49), scatter plots (Fig. S29, S30, S31 and S32, Supplemental Digital Content 14, S41, S42, S43 and S44, Supplemental Digital Content 53), and forest plots (Fig. S33, S34, S35 and S36, Supplemental Digital Content 13, S45, S46, S47 and S48, Supplemental Digital Content 57): confirm the stability of these weak reverse associations, with no single SNP exerting undue influence. Detailed statistical data for the reverse causal associations between CNS tumors and gut microbial taxa are provided in Table S9, Supplemental Digital Content 16, while Table S10, Supplemental Digital Content 17, contains the corresponding details for the reverse relationships between CNS tumors and inflammatory proteins/cytokines. More importantly, none of the 73 microbial taxa or 11 inflammatory proteins causally associated with CNS tumors in forward MR (pFDR < 0.05) overlapped with those affected by CNS tumors in reverse MR. Despite statistical significance, these reverse effects are biologically negligible (OR values close to 1) and nonoverlapping with forward causal targets. Thus, the primary causality is from gut microbiota/inflammatory proteins to CNS tumors, and reverse causality does not undermine our key conclusions.

### 6.9. Mediation analysis

**Figure 4.**
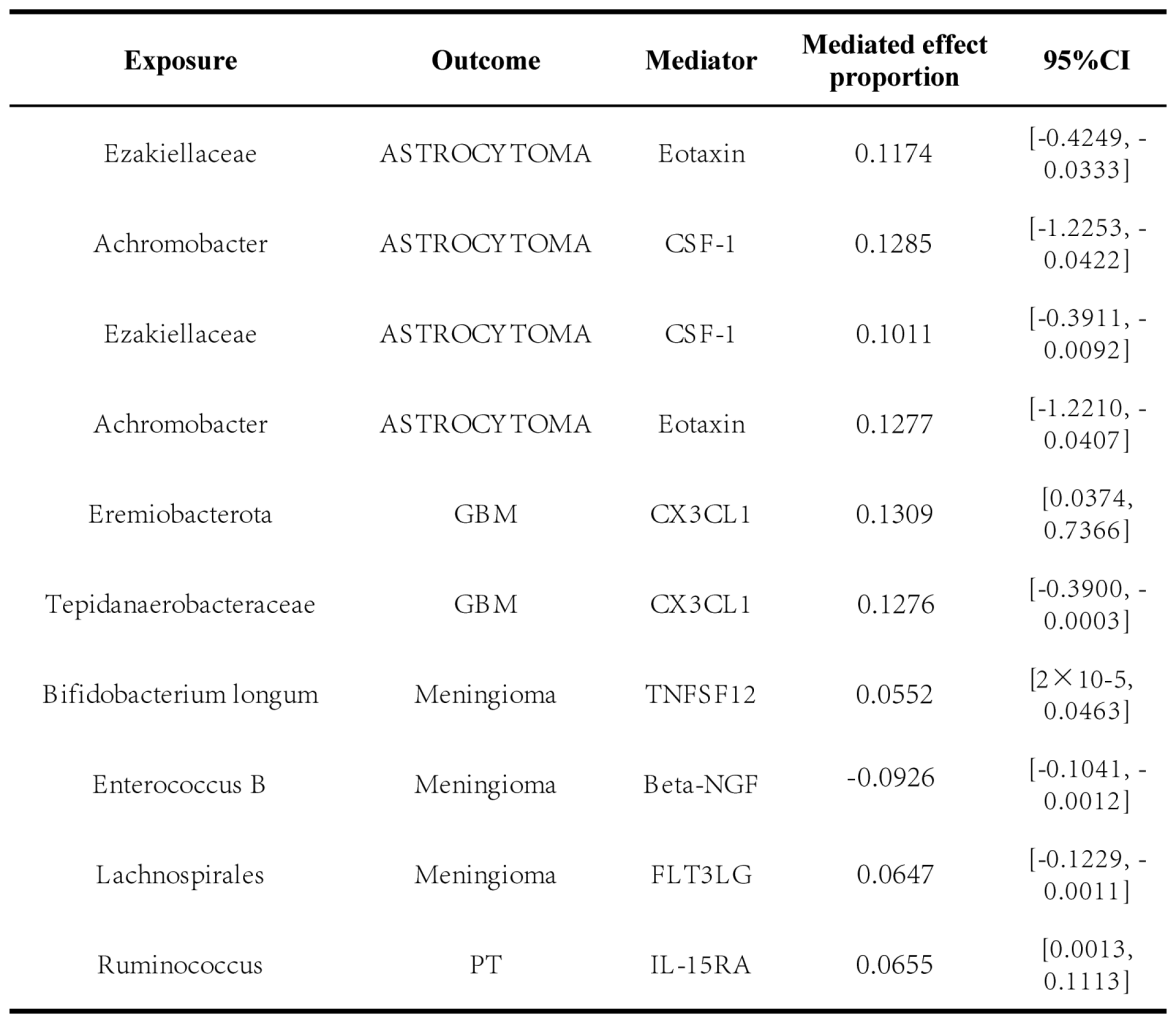
Mediation effects of gut microbiota on CNS tumors via inflammatory proteins. CI = confidence interval.

We further employed a two-step MR framework combined with bootstrap resampling (5000 iterations) to investigate whether circulating inflammatory proteins mediate the pathways from gut microbiota to CNS tumors (Fig. 4), with all detailed mediation effect data, including mediation proportion, 95% CIs, and bootstrap test results, available in Table S11, Supplemental Digital Content 18.

In GBM, CX3CL1 mediated the effects of two gut microbial taxa, with the mediation proportions (indirect effect/total effect) being 0.1309 (95% CI: 0.0374 to 0.7366) for Eremiobacterota and 0.1276 (95% CI: −0.3900 to −0.0003) for Tepidanaerobacteraceae; note that the 95% CI for Eremiobacterota was relatively wide, indicating limited precision of this mediation estimate. For astrocytoma, mediation analysis suggested that Eotaxin may partially mediate the effect of Ezakiellaceae (0.1174, 95% CI: −0.4249 to −0.0333) and Achromobacter (0.1277, 95% CI: −1.2210 to −0.0407), while CSF-1 may partially mediate the effects of Achromobacter (0.1285, 95% CI: −1.2253 to −0.0422) and Ezakiellaceae (0.1011, 95% CI: −0.3911 to −0.0092). For meningioma, multiple cytokines exhibited mediating effects with relatively low estimation precision: TNFSF12 for *Bifidobacterium longum* (0.0552, 95% CI: 0.00002 to 0.0463), Beta-NGF for Enterococcus B (−0.0926, 95% CI: −0.1041 to −0.0012), and FLT3LG for Lachnospirales (0.0647, 95% CI: −0.1229 to −0.0011); the lower bound of the 95% CI for TNFSF12 was extremely close to 0, suggesting a weak mediation signal. In the case of PT, IL-15RA mediated the effect of Ruminococcus with a relatively precise estimate (0.0655, 95% CI: 0.0013 to 0.1113). Notably, several mediation proportions identified in this study exhibited wide 95% CIs (e.g., Eremiobacterota-CX3CL1 in GBM, Achromobacter-Eotaxin/CSF-1 in astrocytoma), which may be attributed to the relatively small number of IVs for the corresponding gut microbial taxa and the inherent heterogeneity of the gut-immune-brain axis. These wide intervals indicate substantial uncertainty in the magnitude of the mediation effects, and the results should be interpreted with caution.

### 6.10. Expression of CX3CL1 in the TME of GBM

**Figure 5.**
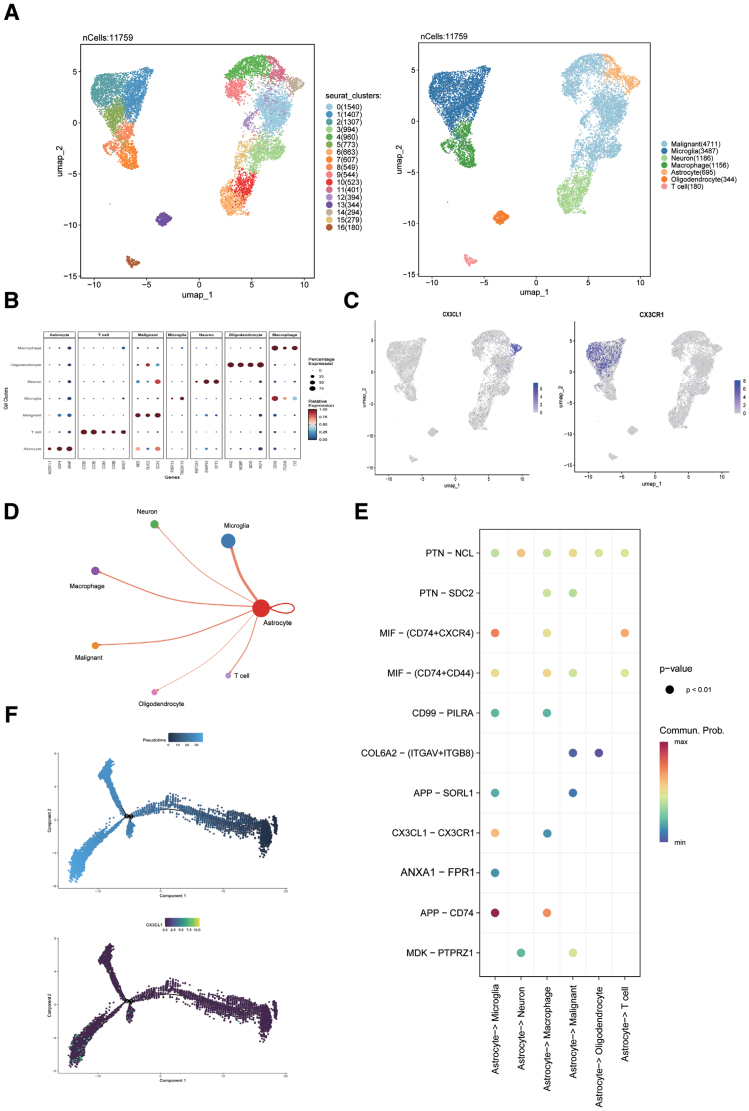
Single-cell transcriptomic profiling reveals CX3CL1 expression and functional relevance in the GBM tumor microenvironment. (A) UMAP projections of 11,759 aggregate single cells from 10 patients showing the composition of different cell types in human gliomas. UMAP projections are shown by cluster numbers and cluster assignment. (B) Bubble plot showing top marker genes for each identified cell cluster. (C) UMAP plots showing the expression distribution of CX3CL1 (left) and its receptor CX3CR1 (right) in the GBM microenvironment. (D) Circos plot depicting cell-cell communication networks originating from astrocytes to other cell types in the GBM microenvironment. (E) Bubble plots showing the expression of selected ligand-receptor pairs mediating communication from astrocytes to other cell subpopulations in gliomas. (F) Pseudo time trajectory analysis of astrocytes in the GBM microenvironment and expression patterns of CX3CL1 during macrophage differentiation. GBM = Glioblastoma.

To clarify the cellular mechanisms involved in CX3CL1-dependent microbial signal transduction in GBM, we conducted single-cell RNA sequencing analysis using the GSE131928_10X dataset, which includes 11,759 cells from 10 human glioma patients. Unsupervised clustering produced 16 distinct cell clusters, which we annotated into 7 primary cell types (Fig. 5A). We validated the accuracy of cell annotations through the marker genes of each corresponding cell population (Fig. 5B). Spatially profiling the expression of CX3CL1 and its receptor CX3CR1 showed that CX3CL1 was significantly enriched in astrocytes, while CX3CR1 was expressed in monocytes and predominantly localized in microglia (Fig. 5C). Our cell-cell communication analysis further pinpointed a robust signaling between astrocytes and microglia (Fig. 5D). Quantitative analysis of ligand-receptor pairs confirmed the existence of CX3CL1-CX3CR1 signaling mediating astrocyte-microglia crosstalk (Fig. 5E). Finally, pseudo time trajectory analysis of astrocytes showed that CX3CL1 expression was upregulated in late-state differentiated astrocytes (Fig. 5F).

Together, these data provide cellular-level support for the MR-inferred potential causal axis linking gut microbiota, CX3CL1 and GBM, offering insights into the possible mechanism by which CX3CL1 may exert its protective role in the unique immune microenvironment of GBM. The immune microenvironment of the CNS is dominated by microglia, which is starkly different from the peripheral immune microenvironment. Our single-cell data support that the primary cellular source of CX3CL1 is astrocytes, and its receptor CX3CR1 is enriched in microglia. Together with our MR evidence that gut microbiota can regulate CX3CL1, this supports a hypothesized cascade where microbial perturbations may induce secretion of CX3CL1 by astrocytes, which in turn binds to CX3CR1 on microglia, potentially driving a distinct astrocyte-microglia crosstalk to reprogram the immune microenvironment of GBM into an antitumor state. These results provide a potential mechanistic framework linking MR-derived causal associations to intra-tumor cellular dynamics, though this putative pathway requires experimental validation. Of note, what specific microbial-derived factor can drive the expression of CX3CL1 in astrocytes and how that alters the functional state of microglia warrants further in-depth experimental investigation.

## 7. Discussion

Gut-immune-brain axis stands at the center stage of novel biological regulatory networks in neurological disorders, but its causal role in CNS tumorigenesis remains ill-defined. Here, we report a multidimensional, systematic and principled analytic framework integrating two-sample MR, mediation analysis and scRNA-seq to interrogate the subtype-specific causal axes between gut microbiota, circulating inflammatory proteins and 4 most common subtypes of CNS tumors. We identified a set of distinct core microbial taxa and inflammatory mediators that were genetically associated with CNS tumor-risk in MR analyze, and that the circulating inflammatory proteins were identified as potential intermediate mediators for several CNS tumor types. These results strengthen causal inference regarding the functional relevance of the gut-immune-brain axis in CNS tumor development, addressing several critical caveats of prior observational studies.

A notable theme that emerged from our study is that well-established microbial taxa perform functions at odds with current literature, which can be explained by the highly context-dependent nature of microbial-host interaction in TMEs. The genus *Bifidobacterium* is widely recognized as probiotic, and multiple strains have been shown to mediate antitumor functions, particularly in CRC, through a variety of inflammatory axes.^[34–36]^ Against this backdrop, our findings demonstrated a clear strain-specific dichotomy in CNS tumors, where *Bifidobacterium kashiwanohense* serves as a risk factor for astrocytoma, *Bifidobacterium ruminantium* and *Bifidobacterium longum* confer a higher odds to meningioma, whereas *Bifidobacterium infantis* is protective against PTs. This paradox can be explained by 2 key inter-related functional drivers: strain-specific metabolic heterogeneity, and stark immunological differences between CRC and CNS TMEs. Beneficial antitumor *Bifidobacterium* strains secrete distinct sets of metabolites (e.g. inosine, short-chain fatty acids) that reinforce the intestinal barrier and induce antitumor immunity against CRC.^[37–39]^ an immunologically accessible central tissue type where direct crosstalk between gut-derived metabolites and mucosa-resident immune cell populations directly determines the tumor outcome. By contrast, *Bifidobacterium kashiwanohense*, *Bifidobacterium ruminantium*, and *Bifidobacterium longum* produce a distinct set of metabolites that modulate neuroinflammation and differentially alter BBB permeability, and unlike the short-chain fatty acids produced by antitumor strains that aid in reinforcing the intestinal barrier, these metabolites can compromise barrier integrity and allow a cascade of pro-inflammatory mediators to breach the CNS,^[40,41]^ though this mechanistic pathway remains inferential and requires experimental confirmation. As an immunoprivileged site, the immune microenvironment of the CNS is dominated by a network of glial cells and peripheral immune infiltrates, which render microbe-induced barrier breach a fundamentally different end phenotype than in CRC. Thus, the combination of strain-specific metabolism and tissue-specific immunology explains how *Bifidobacterium* strains with conclusive antitumor evidence in CRC can play a direct causal role in promoting certain CNS tumors. This highly context-dependent and functionally plastic interplay between bacterial metabolism and immune equilibrium underscores the current limitation of genus-level microbial characterization, and highlights the need for detailed strain-level taxonomical resolution and tissue-specific functional validation in future microbiome-cancer studies.

Our systematic MR analysis of 91 circulating inflammatory proteins uncovered 11 with significant causal associations with CNS tumors, supporting the prevailing notion that chronic inflammation can be a direct driver of tumorigenesis. Moreover, the spectrum of causally relevant mediators has a clear axis of subtype-specific functional divergence, exemplified by an antitumor role of CX3CL1 in GBM, a finding that is further powerfully substantiated by our updated single-cell RNA sequencing data. We determined that CX3CL1 was specifically expressed by astrocytes in the TME of GBM, and that its functional effect ties directly to the crosstalk with oligodendrocytes. This unique pattern of cellular compartmentalization and linkage to functional effect is a direct reflection of the unique immunophysiology of the CNS, where a network of glial cells forms the predominant axis of cellular interaction and directly determines the inflammatory response and tissue homeostasis. By contrast, the pro-tumor role of CX3CL1 reported in other disease settings is tightly linked to its regulatory effect on M1 macrophage polarization.^[42,43]^ This stark contrast provides a definitive demonstration of how tissue-specific cellular crosstalk can completely sway the pro- or antitumor effect of key inflammatory mediators.

Our mediation analysis reveals the responsible subtype-specific inflammatory mediators act as functional connectors linking gut microbes with CNS tumor subtypes. Contrary to the original hypothesis that mediation is unique to GBM, our expanded analysis clearly shows inflammatory mediation is a conserved yet tumor-type-specific feature in all CNS tumor subtypes studied (Table S11, Supplemental Digital Content 18). CX3CL1 was identified as a potential mediator of the effect of *Enteroscipio, Eremiobacterota*, and *Tepidanaerobacteraceae* in GBM, highlighting the pivotal role of the gut-immune-tumor axis in influencing the tumor immune microenvironment to confer protection. Eotaxin and CSF-1 emerge as core mediators in astrocytoma, pointing to a crucial role of innate immune cell recruitment as a central mechanism of gut microbiota-induced tumor progression. The convergence of TNFSF12, Beta-NGF and FLT3LG in mediating gut microbe effects in meningioma suggests complex integration of diverse biological pathways in regulating tumorigenesis, spanning immune activation, neuronal signaling, and granulocyte recruitment.^[44–46]^ IL-15RA mediated microbial effects in the PT correlate with its known involvement in pituitary adenoma pathogenesis.^[47,48]^ Together, these results highlight that the gut-immune-tumor axis acts through distinct, subtype-adaptive signaling pathways in different CNS tumors rather than through a universal mechanism.

The mechanistic framework we delineated is highly consistent with previous gut-brain axis research demonstrating: access of microbial metabolites to the CNS through circulating system^[49]^; chronic low-grade inflammation and increased circulating inflammatory mediators driven by gut dysbiosis^[50]^; and propionate mediated TGF-β signaling upregulated glioma in response to diet-induced microbial perturbation.^[51]^ Despite this consistency, our proposed links between microbiota-derived signals and CNS cytokine modulation remain based on statistical inference rather than direct experimental evidence. These similarities emphasize the potential conserved role of gut microbes in regulating CNS homeostasis, while also highlighting pathogenic mechanisms that are intimately shaped by disease-specific tissue micro environmental features such as BBB permeability and immune cell infiltration signatures in GBM. The exact molecular event cascade underlying the CX3CL1-mediated protection in GBM in particular merits further mechanistic studies.^[52–54]^

As the largest MR to date investigating the causal relationship among gut microbes, inflammatory proteins, and multiple CNS tumor subtypes, our study mitigates some of the key limitations inherited from prior observational studies. A pivotal strength of our study is the integrated analytical framework-two samples MR combined with mediation analysis- which allows for systematic dissection of the “gut microbiota-circulating inflammatory protein-CNS tumor” causal hierarchy, while largely eliminating confounding factors and reverse causality that often mask true causal associations in cross-section studies.

Nonetheless, our results should be interpreted in light of some key limitations. First, our reliance on European GWAS data may introduce population stratification bias, which limits the generalizability of our findings to non-European ethnic populations. Second, genetic proxies for gut microbiota and inflammatory proteins were not able to capture their temporal dynamics in response to environmental factors such as diet and aging. Third, we relied on blood cytokine measurements, which precluded us from directly assessing the impact of CNS-localized inflammatory signaling, a key compartment in brain tumor biology. Fourth, the reverse MR analyses may be limited by statistical power. The smaller number of SNPs available as IVs for CNS tumors (compared to gut microbiota and inflammatory proteins) could restrict the ability to detect robust causal effects, contributing to the modest OR values (close to 1) observed. Future studies with larger GWAS datasets for CNS tumors may help clarify the potential biological relevance of these reverse associations. Fifth, the proposed mechanistic links between microbiota-derived signals, circulating inflammatory proteins, and CNS tumor development remain based on statistical inference from MR and scRNA-seq analyses, rather than direct experimental validation. While our findings are consistent with existing gut-brain axis research, the causal cascade of microbial metabolites → barrier function → cytokine modulation → tumor-risk requires in vitro and in vivo experimental confirmation to rule out alternative pathways and establish biological plausibility. Future studies incorporating multi-ethnic populations, longitudinal study designs and metagenomics sequencing are also needed to confirm the robustness of our findings. Moreover, functional validation of the key microbial taxa and inflammatory mediators in preclinical models will be critical for translating these causal associations into actionable strategies for the prevention and treatment of CNS tumors.

## 8. Conclusion

In this large-scale MR study, we identified genetically predicted causal relationships between gut microbiota, circulating inflammatory proteins, and 4 major CNS tumors. Our study identified 38 gut microbial taxa associated with risk and 35 protective taxa, alongside 5 risk and 6 protective inflammatory proteins. Most importantly, mediation analyses identified inflammatory proteins as potential functional intermediates in the gut-brain tumor axis across the 4 tumors, rather than uniquely in GBM as we initially hypothesized. Mediation analysis suggested that CX3CL1 may partially mediate the effects of specific gut microbes on GBM, while other cytokines, for example, Eotaxin, CSF-1, TNFSF12 and IL-15RA, may exhibit tumor-specific potential mediating roles in astrocytoma, meningioma and PT, respectively. Single-cell evidence further localized CX3CL1 expression primarily to astrocytes and its receptor CX3CR1 to monocytes with predominant expression in microglia within the GBM TME, providing cellular-level evidence of its role in astrocyte-microglia crosstalk and antitumor immune regulation.

## Author contributions

**Data curation:** Xianwen Cao, Kun Wang, Hui Guo, Qiang Wu.

**Formal analysis:** Xianwen Cao, Junfei Shao.

**Methodology:** Xianwen Cao, Kun Wang, Hui Guo.

**Resources:** Xianwen Cao, Hui Guo.

**Validation:** Xianwen Cao.

**Visualization:** Xianwen Cao, Xuhan Wang.

**Conceptualization:** Kun Wang, Junfei Shao.

**Investigation:** Kun Wang.

**Software:** Kun Wang, Hui Guo.

**Supervision:** Hui Guo, Junfei Shao.

**Funding acquisition:** Junfei Shao.

**Project administration:** Junfei Shao.

**Writing – original draft:** Xianwen Cao, Kun Wang.

**Writing – review & editing:** Junfei Shao.


























































































































