# Supplementary material for: The gut-immune-brain axis in CNS tumors: Causal roles of microbiota and inflammatory proteins unveiled by Mendelian randomization and single-cell transcriptomics
Source: Medicine (Baltimore). 2026 Aug 7;105(32):e50109. doi: 10.1097/MD.0000000000050109 (PMC13456912; doi:10.1097/MD.0000000000050109)
Supplement: Supplementary file 1 [file medi-105-e50109-s001.docx]

**STROBE-MR checklist of recommended items to address in reports of Mendelian randomization studies**^1^ ^2^

| **Item No.** | **Section** | **Checklist item** | **Page No.** | **Relevant text from manuscript** |
| --- | --- | --- | --- | --- |
| 1 | **TITLE and ABSTRACT** | Indicate Mendelian randomization (MR) as the study’s design in the title and/or the abstract if that is a main purpose of the study | 1 | The Gut-Immune-Brain Axis in CNS Tumors: Causal Roles of Microbiota and Inflammatory Proteins Unveiled by Mendelian Randomization and Single-Cell Transcriptomics |
|  | **INTRODUCTION** |  |  |  |
| 2 | **Background** | Explain the scientific background and rationale for the reported study. What is the exposure? Is a potential causal relationship between exposure and outcome plausible? Justify why MR is a helpful method to address the study question | 2/3 | There is a growing number of research suggesting that there is an association between gut microbiota and central nervous system (CNS) tumor. However, the causal relationships and the mediation effects of inflammatory proteins in the associations are unclear.  MR is a commonly used analytical method in the field of genetic epidemiology. In MR studies, researchers use genetic variations as instrumental variables (IVs) to assess whether there is a causal effect between exposure factors and outcomes, as well as the magnitude of such an effect.They facilitate causal inference through genetic proxies, while addressing constraints associated with sample size and measurement accuracy |
| 3 | **Objectives** | State specific objectives clearly, including pre-specified causal hypotheses (if any). State that MR is a method that, under specific assumptions, intends to estimate causal effects | 3 | In this research, a two-sample MR approach was applied to thoroughly investigate the causal associations between 473 gut microbial traits, 91 circulatory inflammatory proteins, and four CNS tumors. We perform mediation analysis to examine whether inflammatory proteins are mediators in the gut microbiota–tumor axis. |
|  | **METHODS** |  |  |  |
| 4 | **Study design and data sources** | Present key elements of the study design early in the article. Consider including a table listing sources of data for all phases of the study. For each data source contributing to the analysis, describe the following: |  |  |
|  | a) | Setting: Describe the study design and the underlying population, if possible. Describe the setting, locations, and relevant dates, including periods of recruitment, exposure, follow-up, and data collection, when available. | 4/5 | All these GWAS data—spanning the accession ranges from GCST90032172 to GCST90032644 for gut microbiota and from GCST90274758 to GCST90274848 for inflammatory proteins—are openly accessible in the NHGRI - EBI Catalog.  In order to yield an adequate SNP panel for downstream analyses, we selected gut microbiota-related SNPs with a threshold of P < 1×10⁻⁵. Second, linkage disequilibrium (LD) loci were excluded and we retained gut microbiota-related SNPs with strict LD thresholds as follows: pairwise correlation coefficient r2 < 0.001, physical distance> 10,000 kb . |
|  | b) | Participants: Give the eligibility criteria, and the sources and methods of selection of participants. Report the sample size, and whether any power or sample size calculations were carried out prior to the main analysis | 5 | Further specifics are outlined in Table S2. |
|  | c) | Describe measurement, quality control and selection of genetic variants | 5 | We selected gut microbiota-related SNPs with a threshold of P < 1×10⁻⁵.Linkage disequilibrium (LD) loci were excluded... pairwise correlation coefficient r² < 0.001, physical distance > 10,000 kb.Palindromic SNPs were excluded; in other words, we eliminated SNPs with A/T or G/C alleles.Finally, the explained variance (R²) and F-statistics were calculated... If the F-statistics of a SNP is less than 10, it can be considered a weak instrument.We utilized Benjamini-Hochberg False Discovery Rate (FDR) correction for multiple testing, and FDR-adjusted p-values (pFDR) < 0.05 were considered statistically significant. |
|  | d) | For each exposure, outcome, and other relevant variables, describe methods of assessment and diagnostic criteria for diseases | 5 | Operating as a prospective cohort investigation, the FinnGen consortium pinpointed CNS tumor cases by utilizing ICD diagnostic codes. Genetic information corresponding to each of the four CNS tumor categories was separately acquired from the FinnGen database. |
|  | e) | Provide details of ethics committee approval and participant informed consent, if relevant | 5 | This study was a secondary analysis of publicly available GWAS summary statistics. Ethical approval was obtained for all original GWAS studies. Notably, because this study did not include any individual-level data, no additional approval from an ethics committee was needed. |
| 5 | **Assumptions** | Explicitly state the three core IV assumptions for the main analysis (relevance, independence and exclusion restriction) as well assumptions for any additional or sensitivity analysis | 5 | MR analysis satisfied three main assumptions: (1) IVs were strongly associated with exposures; (2) IVs had no association with confounders of exposure–outcome associations; (3) IVs had no direct influence on study outcomes and only affected study outcomes via exposures. |
| 6 | **Statistical methods: main analysis** | Describe statistical methods and statistics used |  |  |
|  | a) | Describe how quantitative variables were handled in the analyses (i.e., scale, units, model) |  | NA |
|  | b) | Describe how genetic variants were handled in the analyses and, if applicable, how their weights were selected | 5 | In order to yield an adequate SNP panel for downstream analyses, we selected gut microbiota-related SNPs with a threshold of P < 1×10⁻⁵. Second, ... pairwise correlation coefficient r2 < 0.001, physical distance> 10,000 kb [17]. Last, ... After harmonizing the outcome data, palindromic SNPs were excluded; in other words, we eliminated SNPs with A/T or G/C alleles. We also collected related details from every SNP: the position on the chromosome, the effect allele (EA), the non-effect allele (OA), the effect allele frequency (EAF), the effect (β), the standard error for β (SE), and the corresponding P-value. Finally, the explained variance (R2) and F-statistics were calculated to ensure solid correlations between all IVs and the exposure. If the F-statistics of a SNP is less than 10, it can be considered a weak instrument [18]. |
|  | c) | Describe the MR estimator (e.g. two-stage least squares, Wald ratio) and related statistics. Detail the included covariates and, in case of two-sample MR, whether the same covariate set was used for adjustment in the two samples | 5 | For main MR analyses of the causal effects of gut microbiota/inflammatory proteins on CNS tumors, we conducted two-sample MR analysis (detailed in Steps 1A and 2A; Fig. 1) using five approaches: Inverse Variance Weighted (IVW)[19], Weighted Mode[20], MR-Egger [21], Weighted Median [22], and Simple Mode [23]. In these steps, the IVW method was taken as the principal one, and results are reported as odds ratios (ORs) and 95% confidence intervals (CIs). The IVW P-value threshold determined statistical significance. Our results were considered statistically significant based on the following two criteria: 1) the IVW P-value was less than 0.05, and 2) the results of IVW and MR-Egger were directionally concordant. |
|  | d) | Explain how missing data were addressed |  | NA |
|  | e) | If applicable, indicate how multiple testing was addressed |  | NA |
| 7 | **Assessment of assumptions** | Describe any methods or prior knowledge used to assess the assumptions or justify their validity | 5/6 | Finally, we calculated the explained variance (R²) and F-statistics to validate robust correlations between instrumental variables (IVs) and the exposure. SNPs with F-statistics less than 10 were classified as weak instruments[18].  We performed three types of sensitivity analyses.: ... Third, we applied MR-PRESSO and MR-Egger regression methods to identify and correct for horizontal pleiotropy [25, 26]. |
| 8 | **Sensitivity analyses and additional analyses** | Describe any sensitivity analyses or additional analyses performed (e.g. comparison of effect estimates from different approaches, independent replication, bias analytic techniques, validation of instruments, simulations) | 6 | We performed three types of sensitivity analyses. First, we used Cochran’s Q tests to assess heterogeneity [24] between causal estimates derived from different SNPs, and scatterplots to graphically view SNPs, exposure, and outcome. Second, leave-one-out analyses were performed by excluding each alternative SNP and recalculating the results using the remaining SNPs. Third, we applied MR-PRESSO and MR-Egger regression methods to identify and correct for horizontal pleiotropy [25, 26]. In particular, we used MR-PRESSO outlier tests for the detection of horizontal pleiotropy by excluding outlier SNPs. Furthermore, we utilized Benjamini-Hochberg False Discovery Rate (FDR) correction for multiple testing, and FDR-adjusted p-values (pFDR) < 0.05 were considered statistically significant. |
| 9 | **Software and pre-registration** |  |  |  |
|  | a) | Name statistical software and package(s), including version and settings used | 6 | All analyses were executed in R version v4.3.2. The "TwoSampleMR" package was utilized for Mendelian randomization analyses, and "MRPRESSO" was used for pleiotropy assessment. |
|  | b) | State whether the study protocol and details were pre-registered (as well as when and where) |  | NA |
|  | **RESULTS** |  |  |  |
| 10 | **Descriptive data** |  |  |  |
|  | a) | Report the numbers of individuals at each stage of included studies and reasons for exclusion. Consider use of a flow diagram |  | NA |
|  | b) | Report summary statistics for phenotypic exposure(s), outcome(s), and other relevant variables (e.g. means, SDs, proportions) |  | NA |
|  | c) | If the data sources include meta-analyses of previous studies, provide the assessments of heterogeneity across these studies |  | NA |
|  | d) | For two-sample MR:  i.  Provide justification of the similarity of the genetic variant-exposure associations between the exposure and outcome samples  ii.  Provide information on the number of individuals who overlap between the exposure and outcome studies |  | NA |
| 11 | **Main results** |  |  |  |
|  | a) | Report the associations between genetic variant and exposure, and between genetic variant and outcome, preferably on an interpretable scale | 5 | In order to yield an adequate SNP panel for downstream analyses, we selected gut microbiota-related SNPs with a threshold of P < 1×10⁻⁵. Second, linkage disequilibrium (LD) loci were excluded and we retained gut microbiota-related SNPs with strict LD thresholds as follows: pairwise correlation coefficient r2 < 0.001, physical distance> 10,000 kb [17]. Fig. 2 |
|  | b) | Report MR estimates of the relationship between exposure and outcome, and the measures of uncertainty from the MR analysis, on an interpretable scale, such as odds ratio or relative risk per SD difference |  | Fig .2 and Fig .3 |
|  | c) | If relevant, consider translating estimates of relative risk into absolute risk for a meaningful time period |  | NA |
|  | d) | Consider plots to visualize results (e.g. forest plot, scatterplot of associations between genetic variants and outcome versus between genetic variants and exposure) |  | Fig. 2-4  Figures S1-45 |
| 12 | **Assessment of assumptions** |  |  |  |
|  | a) | Report the assessment of the validity of the assumptions | 4 | MR analysis satisfied three main assumptions: (1) IVs were strongly associated with exposures; (2) IVs had no association with confounders of exposure–outcome associations; (3) IVs had no direct influence on study outcomes and only affected study outcomes via exposures. |
|  | b) | Report any additional statistics (e.g., assessments of heterogeneity across genetic variants, such as *I^2^*, Q statistic or E-value) | 6 | Cochran’s Q tests revealed no significant heterogeneity (P > 0.05).MR-Egger regression intercept tests showed no signs of directional pleiotropy (P > 0.05).MR-PRESSO analyses confirmed that horizontal pleiotropy was absent (P > 0.05).Leave-one-out analyses validated the stability of the results, as the null line lay outside the overall confidence intervals. |
| 13 | **Sensitivity analyses and additional analyses** |  |  |  |
|  | a) | Report any sensitivity analyses to assess the robustness of the main results to violations of the assumptions | 8 | MR-Egger regression intercept tests showed no signs of directional pleiotropy (P > 0.05). At the same time, supplementary evidence that horizontal pleiotropy does not exist was found in the MR-PRESSO analyses (P > 0.05), and all related details are displayed in Table S8. Furthermore, the results of Cochran's Q tests, which are also documented in Table S8, indicated that there was no statistically significant heterogeneity (P > 0.05).By using the Benjamini-Hochberg false discovery rate (FDR) correction, the IVW results were adjusted for multiple testing. All of the aforementioned significant connections remained robust following the FDR adjustment (pFDR <0.05), and all related factual statistics are listed in Table S8. |
|  | b) | Report results from other sensitivity analyses or additional analyses | 5 | using five approaches: Inverse Variance Weighted (IVW)[19], Weighted Mode[20], MR-Egger [21], Weighted Median [22], and Simple Mode [23]. |
|  | c) | Report any assessment of direction of causal relationship (e.g., bidirectional MR) | 9 | Despite statistical significance, these reverse effects are biologically negligible (OR values close to 1) and non-overlapping with forward causal targets. Thus, the primary causality is from gut microbiota/inflammatory proteins to CNS tumors, and reverse causality does not undermine our key conclusions. |

|  | d) | When relevant, report and compare with estimates from non-MR analyses | 7/8 | Previous studies confirmed Achromobacter, enriched by metformin, enhances anti-tumor effects in colorectal cancer models [27], supporting its protective role.Providencia (an opportunistic pathogen) was reported to impair intestinal barrier function and increase permeability [28], consistent with its tumor-promoting effect here...Gemmatimonadaceae has been reported to show increased abundance in the vagina of cervical cancer patients undergoing radiation therapy... and its DNA was detected in lung cancer patients... This aligns with its strong tumor-promoting effect in GBM observed in our study......its congener Fusobacterium nucleatum has been well-documented to promote the development of CRC [33]... The tumor-promoting effect of Fusobacterium A on meningioma observed in our study provides a novel extension... |
| --- | --- | --- | --- | --- |
|  | e) | Consider additional plots to visualize results (e.g., leave-one-out analyses) |  | Figures S1-S23 |
|  | **DISCUSSION** |  |  |  |
| 14 | **Key results** | Summarize key results with reference to study objectives | 12 | In this large-scale MR study, we identified genetically predicted causal relationship between gut microbiota, circulating inflammatory proteins, and four major CNS tumors. Our study identified 38 gut microbial taxa associated with risk and 35 protective taxa, alongside 5 risk and 6 protective inflammatory proteins. |
| 15 | **Limitations** | Discuss limitations of the study, taking into account the validity of the IV assumptions, other sources of potential bias, and imprecision. Discuss both direction and magnitude of any potential bias and any efforts to address them | 12 | Nonetheless, our results should be interpreted in light of some key limitations. First ... and treatment of CNS tumors. |
| 16 | **Interpretation** |  |  |  |
|  | a) | Meaning: Give a cautious overall interpretation of results in the context of their limitations and in comparison with other studies | 10 | A notable theme that emerged from our study is that well-established microbial taxa perform functions at odds with current literature, which can be explained by the highly context-dependent nature of microbial-host interaction in tumor microenvironments. |
|  | b) | Mechanism: Discuss underlying biological mechanisms that could drive a potential causal relationship between the investigated exposure and the outcome, and whether the gene-environment equivalence assumption is reasonable. Use causal language carefully, clarifying that IV estimates may provide causal effects only under certain assumptions | 12 | The mechanistic framework we delineated is highly consistent with previous gut–brain axis research demonstrating: access of microbial metabolites to the CNS through circulating system; chronic low-grade inflammation and increased circulating inflammatory mediators driven by gut dysbiosis... |
|  | c) | Clinical relevance: Discuss whether the results have clinical or public policy relevance, and to what extent they inform effect sizes of possible interventions | 11 | This highly context dependent and functionally plastic interplay ... highlights the need for detailed strain-level taxonomical resolution and tissue specific functional validation in future microbiome-cancer studies |
| 17 | **Generalizability** | Discuss the generalizability of the study results (a) to other populations, (b) across other exposure periods/timings, and (c) across other levels of exposure | 12 | First, our reliance on European GWAS data may introduce population stratification bias, which limits the generalizability of our findings to non-European ethnic populations. |
|  | **OTHER INFORMATION** |  |  |  |
| 18 | **Funding** | Describe sources of funding and the role of funders in the present study and, if applicable, sources of funding for the databases and original study or studies on which the present study is based | 13 | This work was supported by the Wuxi Taihu Lake Talent Plan - Supports for Leading Talents in Medical and Health Profession (2020THRC-DJ-SNW), the Key Project of the Jiangsu Provincial Health Commission (ZD2022038), and the General Project of the Wuxi Medical Center, Nanjing Medical University (WMCG202402). |
| 19 | **Data and data sharing** | Provide the data used to perform all analyses or report where and how the data can be accessed, and reference these sources in the article. Provide the statistical code needed to reproduce the results in the article, or report whether the code is publicly accessible and if so, where | 4 | GWAS data on gut microbiota was derived from the dataset published by Kurilshikov et al., which includes 473 gut microbial features and is based on 18,340 individuals [15]. Data on 91 circulating inflammatory proteins came from a meta-analysis covering 11 cohorts, involving a total of 14,824 European participants [16]. All these GWAS data—spanning the accession ranges from GCST90032172 to GCST90032644 for gut microbiota and from GCST90274758 to GCST90274848 for inflammatory proteins—are openly accessible in the NHGRI-EBI Catalog. |
| 20 | **Conflicts of Interest** | All authors should declare all potential conflicts of interest | 13 | The authors claim that we performed this research without any business or financial relationships that could be interpreted as potential conflicts of interest |

This checklist is copyrighted by the Equator Network under the Creative Commons Attribution 3.0 Unported (CC BY 3.0) license.

1.	Skrivankova VW, Richmond RC, Woolf BAR, Yarmolinsky J, Davies NM, Swanson SA, et al. Strengthening the Reporting of Observational Studies in Epidemiology using Mendelian Randomization (STROBE-MR) Statement. JAMA. 2021;under review.

2.	Skrivankova VW, Richmond RC, Woolf BAR, Davies NM, Swanson SA, VanderWeele TJ, et al. Strengthening the Reporting of Observational Studies in Epidemiology using Mendelian Randomisation (STROBE-MR): Explanation and Elaboration. BMJ. 2021;375:n2233.
